# Proteomic investigation of protein adsorption to cerebral microdialysis membranes in surgically treated intracerebral hemorrhage patients - a pilot study

**DOI:** 10.1186/s12953-020-00163-7

**Published:** 2020-07-25

**Authors:** Lovisa Tobieson, Zita Czifra, Karin Wåhlén, Niklas Marklund, Bijar Ghafouri

**Affiliations:** 1 Department of Neurosurgery in Linköping, and Department of Biomedical and Clinical Sciences, Linköping University, University Hospital, SE-581 85 Linköping, Sweden; 2 Pain and Rehabilitation Center, and Department of Health, Medicine and Caring Sciences, Linköping University, Linköping, Sweden; 3 Department of Clinical Sciences Lund, Lund University, Skåne University Hospital, Neurosurgery, Lund, Sweden

**Keywords:** Cerebral microdialysis, Intracerebral hemorrhage, Proteomics, Biomarker, Protein adsorption, Relative recovery

## Abstract

**Background:**

Cerebral microdialysis (CMD) is a minimally invasive technique for sampling the interstitial fluid in human brain tissue. CMD allows monitoring the metabolic state of tissue, as well as sampling macromolecules such as proteins and peptides. Recovery of proteins or peptides can be hampered by their adsorption to the CMD membrane as has been previously shown in-vitro*,* however, protein adsorption to CMD membranes has not been characterized following implantation in human brain tissue.

**Methods:**

In this paper, we describe the pattern of proteins adsorbed to CMD membranes compared to that of the microdialysate and of cerebrospinal fluid (CSF). We retrieved CMD membranes from three surgically treated intracerebral hemorrhage (ICH) patients, and analyzed protein adsorption to the membranes using two-dimensional gel electrophoresis (2-DE) in combination with nano-liquid mass spectrometry. We compared the proteome profile of three compartments; the CMD membrane, the microdialysate and ventricular CSF collected at time of CMD removal.

**Results:**

We found unique protein patterns in the molecular weight range of 10–35 kDa for each of the three compartments.

**Conclusion:**

This study highlights the importance of analyzing the membranes in addition to the microdialysate when using CMD to sample proteins for biomarker investigation.

## Introduction

Sampling, detecting and analyzing protein biomarkers associated with ongoing brain injury is of growing interest in clinical neuroscience research, both in the acute setting of e.g. traumatic brain injury or intracerebral hemorrhage (ICH) as well as in chronic neurodegenerative diseases. Such protein biomarkers may help clarify pathophysiological mechanisms of ongoing brain injury, or guide interventions and aid in prevention of further injury.

There are a number of different methods for sampling biomarkers for neuroproteomic analysis. One method is the collection of cerebrospinal fluid (CSF), either from an external ventricular drain (EVD) used to monitor intracranial pressure (ICP) or from a lumbar puncture. CSF has the advantage of being relatively easy to obtain but does not entirely reflect the interstitial fluid [1, 2]. Another method for sample collection, which does indeed sample the interstitial fluid, is minimally invasive monitoring of brain tissue by cerebral microdialysis (CMD) used since the 1990s for monitoring brain metabolism as part of multimodal monitoring in the neurocritical care setting [3–5]. With the introduction of high-molecular-weight cut-off catheters (100 kDa) it has become possible to sample macromolecules from the interstitial fluid. Thus apart from providing information on brain metabolism, CMD can now also be used for biomarker discovery or determining concentration of specific proteins or peptides of interest.

When using CMD to sample macromolecules from interstitial fluid there are several technical factors to be aware of. The CMD technique involves inserting a thin dialysis catheter consisting of a semipermeable membrane into the brain tissue. The catheter is perfused with fluid which is subsequently collected and analyzed. The collected fluid, referred to as the microdialysate, is a reflection of the interstitial fluid. However, the relative recovery of molecules in the microdialysate is always a fraction of their true tissue concentration and is affected by a variety of factors. These factors include perfusate flow rate and composition; tissue temperature, pH and tortuosity; and protein molecular weight, three dimensional structure and hydrophobicity [6–8]. Furthermore, CMD membrane characteristics such as material, length and diameter, and pore size also affect protein interaction with the membrane surface and thereby recovery [9]. Proteins adsorb to solid surfaces [10] and CMD membranes are no exception. This adsorption may prevent proteins from passing over to the microdialysate, or may cause them to pass over in an unpredictable manner. Protein adsorption may also change over time as the surface gets saturated, which in turn also affects the diffusion of other particles or proteins across the membrane, [7] thereby affecting their relative recovery.

Although protein adsorption to microdialysis membranes has been shown in vitro [6, 8, 11] and in in-vivo in skin [12] it has not been characterized on microdialysis membranes implanted in human brain tissue.

By exploring which proteins adhere to the membrane the protein expression in the interstitial fluid can be characterized more accurately. If only the microdialysate is analyzed and not the membrane, valuable information may be overlooked and important biomarkers might evade detection or the tissue concentration of them may be greatly underestimated. In this pilot study, we aimed to determine if there was a difference in proteome profile of CSF, cerebral microdialysate, and on CMD membranes following implantation by using two-dimensional gel electrophoresis (2-DE) to separate proteins followed by identification by liquid chromatography tandem mass spectrometry (LC-MS/MS).

## Materials and methods

### Patients

Patients undergoing acute neurosurgery for ICH followed by subsequent treatment at the neurocritical care (NCC) unit, University Hospital, Linköping were prospectively recruited to this observational study.

**Fig. 1:**
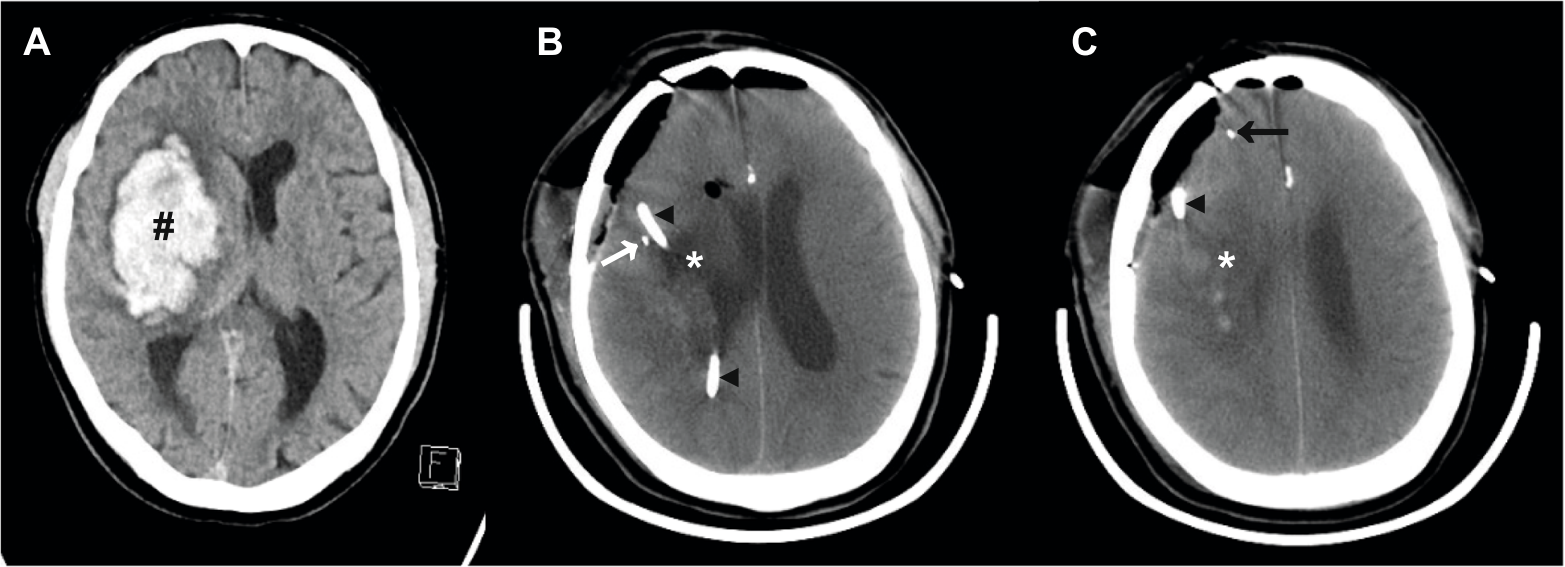
**a** Preoperative Computed Tomography (CT) scan of patient 1 showing the intracerebral hemorrhage (#). **b**-**c** Post-operative CT scan showing the hematoma cavity (*), the perihemorrhagic microdialysis catheter (PHZ; white arrow), **c** the catheter in seemingly normal cortex (SNX; black arrow) and the external ventricular drain (black arrow head;◄)

Patients received dual microdialysis catheters with a molecular weight cut-off of 100 kDa, and membrane length 10 mm (71 High Cut-off Brain MD Catheter, M-dialysis AB, Solna, Sweden) at the time of surgical evacuation of ICH. One catheter was placed within 1 cm of the evacuated hematoma, in the perihemorrhagic zone (PHZ), and the other at a distance of at least 1 cm from the evacuated ICH in a non-eloquent area of seemingly normal brain cortex (SNX) on the ipsilateral side (Fig. 1a-c). Microdialysis sampling was initiated directly post-surgery and microdialysis vials were changed every two hours. The catheters were perfused with a commercially available 5% Human Albumin in a water solution containing the excipients sodium chloride, N-acetyl-DL-tryptophan and caprylic acid (Albunorm, Octapharma, Stockholm, Sweden), at a rate of 0.3 μL/min. The use of albumin in the perfusate, to counteract ultrafiltration and subsequent fluid loss to the tissue, has been clinical routine in our department since 2013. Microdialysate was collected at a time corresponding to 72 h after ICH onset. The results of 2-DE of this microdialysate has been previously published [13].

Patients received an external ventricular drain (EVD) for ICP monitoring from which CSF was collected at a time point corresponding to removal of the microdialysis membranes, towards the end of the patient’s NCC unit treatment period. The 2 first mL of aspired CSF were discarded and the following 2 mL were immediately sent to the laboratory where the sample was centrifuged for 10 min at 1800 x g at 4 °C, and the supernatant was subsequently stored at − 86 °C awaiting analysis.

Microdialysis catheter membranes were removed at the end of the patients’ NCC treatment period, cut directly into a polypropylene tube and stored initially at − 20 °C, then at − 86 °C, awaiting analysis. Three membranes from the perihemorrhagic zone (PHZ1, PHZ2, PHZ3) and two membranes from seemingly normal brain tissue (SNX1, SNX2) were used. The third SNX membrane was accidentally discarded in the NCC unit and thus not available for analysis.

### Sample preparation

#### Preparation of dialysate samples

The proteomic analysis of the microdialysate of the three patients has been previously published [13] along with a detailed description of the sample preparation. Briefly, 40 μL of each sample was applied onto an Albumin & IgG Depletion column (GE Healthcare, Uppsala, Sweden), before being desalted and lyophilized. Protein concentration was determined [14] before applying the sample to 2-DE.

#### Preparation of CSF samples

The CSF samples were applied to albumin and IgG depletion columns (Albumin & IgG Depletion SpinTrap, GE Healthcare) in order to improve detection of low abundant proteins. Samples were then desalted with Amicon Ultra Centrifugal Filters, 0.5 mL Ultracel 3 k (Merck Millipore Ltd., Cork, Ireland), and dried in SAVANT SPD 111 V SpeedVac Concentrator. The dried samples were resolved in 150 μL urea buffer solution (8 M Urea, 4% (w/v) CHAPS, 65 mM DTT, 2% (v/v) pharmalyte 3–10, trace of bromophenol blue) and incubated for 1 h at room temperature before protein concentration was determined by 2-D Quant Kit (GE Healthcare).

#### Preparation of microdialysis membranes

CMD membranes were cut into small pieces and proteins were extracted by adding 100 μL urea buffer solution (8 M Urea, 4% (w/v) CHAPS, 65 mM DTT, 2% (v/v) pharmalyte 3–10, trace of bromophenol blue) and incubating membrane pieces at room temperature with gentle shaking following centrifugation. Protein concentration was determined by 2-D Quant Kit (GE Healthcare) in accordance with standard protocol. An unused 71 High Cut-off Brain MD Catheter (M-Dialysis AB) membrane was used as a blank control.

#### Two-dimensional gel electrophoresis (2-DE)

50 μg protein from each sample was separated on 2-DE [15] and visualized by silver staining [16]. The protein pattern was analyzed as digitized image. The amount of protein in a spot was assessed as background corrected optical density, integrated over all pixels in the spot and expressed as integrated optical density (IOD).

#### Protein identification

Selected protein spots were excised from the gels using a home-made spot picker and destained. Briefly, after removal of water, 25 μL of solution A (30 mM potassium ferricyanide, MilliQ water) and solution B (100 mM sodium thiosulphate pentahydrate, MilliQ water) were added to the gel pieces at the same time to decolor the gel. Gel pieces were then washed 6 × 5 min, incubated at room temperature for 20 min in 50 μL of solution C (200 mM ammonium bicarbonate, MilliQ water) followed by washing 3 × 5 min.

Gel pieces were then dehydrated using 100 μL of 100% acetonitrile (ACN) applied twice. After removing ACN the samples were dried in SpeedVac for 15 min. 10 μL trypsin (200 μg/mL) was mixed with 90 μL 25 mM ammonium bicarbonate (ABC), and 25 μL of this mixture was added to the gel pieces, incubated on ice for at least 30 min to reduce the autolytic activity of trypsin, and then incubated at 37 °C overnight. The supernatant was transferred to a new tube and dried in SpeedVac. Gel pieces were incubated in 40 μL 50% ACN/5% trifluoroacetic acid (TFA) for 3–4 h with gentle shaking to enable further extraction of remaining peptides in the gel pieces. The supernatant obtained from extraction with ACN/TFA was then pooled with the dried peptides and completely dried by SpeedVac, and stored at − 20 °C until further analysis.

Protein identification was initially done using Matrix-assisted laser desorption/ionization time-of-flight mass spectrometry (MALDI-TOF-MS), and low abundant proteins were analyzed using liquid chromatography tandem mass spectrometry (LC-MS/MS). For MALDI analysis the dried peptides were dissolved in 4 μL of 0.1% TFA and 1 μl was mixed with 1 μl of matrix solution (0.067 g/ml 2, 5-dihydroxybenzoic acid (DHB) in 70% acetonitrile, 0.3% TFA). One μL was applied on the MALDI plate. Peptide calibration standard mixture II (Bruker Daltonics) was mixed with 0.1% TFA in a ratio of 1:50 and 1 μl was mixed with DHB 1:1. One μL of mixed standard was applied on the MALDI plate next to each sample. Peptide analysis was then performed in the range of 300–3500 Da using MALDI-TOF-MS (Voyager-DE PRO, Applied Biosystems).

Protein identification for the low abundant proteins was performed using LC-MS/MS whereby the trypsinated peptides were dissolved in 6 μL of 0.1% formic acid (FA) and applied to a nano-flow HPLC system, EASY-nLC II (Thermo Scientific) in conjugation with the mass spectrometer, LTQ Orbitrap Velos Pro hybrid mass spectrometer (Thermo Scientific) with a nano-electrospray source as previously described [17].

Database searching was performed using MS-Fit search engine and MaxQuant version 1.5 with trypsin as digestion enzyme against a human taxonomy of the SwissProt and the NCBI databases. The following search parameters were used: maximum two missed cleavages; fragment ion mass tolerance 0.5 Da; parent ion mass tolerance 6 ppm; fixed modification- carbamidomethylation of cysteine; variable modifications - N-terminal acetylation and methionine oxidation. Data was filtered at 1% false discovery rate. Identifications were based on a minimum of two unique peptides.

### Statistical analysis

Central tendency and dispersion of data are presented as mean and standard deviation when data are summarized; alternatively numbers are presented comprehensively for clarity.

## Results

Microdialysate samples from six catheters, five CMD membranes and three CSF samples were available for proteomic analysis from three ICH patients. CMD membranes were implanted in each patient for 84, 98 and 170 h respectively (Table 1). Total protein concentration (μg/μL) was 0.89 (0.67) in the membrane samples, 1.31 (0.34) in the CSF and 2.37 (1.33) in the dialysate samples. The protein pattern in the dialysate samples was dominated by high amount of albumin despite the attempts to deplete albumin, thus causing a poor resolution of high molecular weight proteins. Therefore the 2-DE images of all compartments were cropped and only protein spots in the area 10–50 kDa were included in analysis. The original 2-DE images are available in supplemental material (Supplemental Figure 1).

**Table 1:** Patient characteristics

*Patient number*	*1*	*2*	*3*
Age (years)	68	55	48
ICH volume (mL)	90	87	57
ICH location and side	BG/R	BG/L	BG/R
Dist. PHZ-MD (mm)	3	7	5
Dist. SNX-MD (mm)	34	13	13
GCS-M on arrival	5	5	5
LOS in NCC (d)	5	5	7
Outcome (mRS)	4	6	4
Time ICH onset to start of sampling (h)	14	16	10
Sampling duration (h)	98	84	170

*Abbreviations*: *ICH* intracerebral hemorrhage, *BG* basal ganglia, *R* right, *L* left, *Dist. PHZ-MD* distance from microdialysis catheter in perihemorrhagic zone to evacuated ICH, *Dist. SNX-MD* distance from microdialysis catheter in seemingly normal cortex to evacuated ICH, *GCS-M* Glasgow Coma Scale Motor score, *LOS* length of stay, *NCC* neurocritical care, *mRS* modified Rankin Scale

**Fig. 2:**
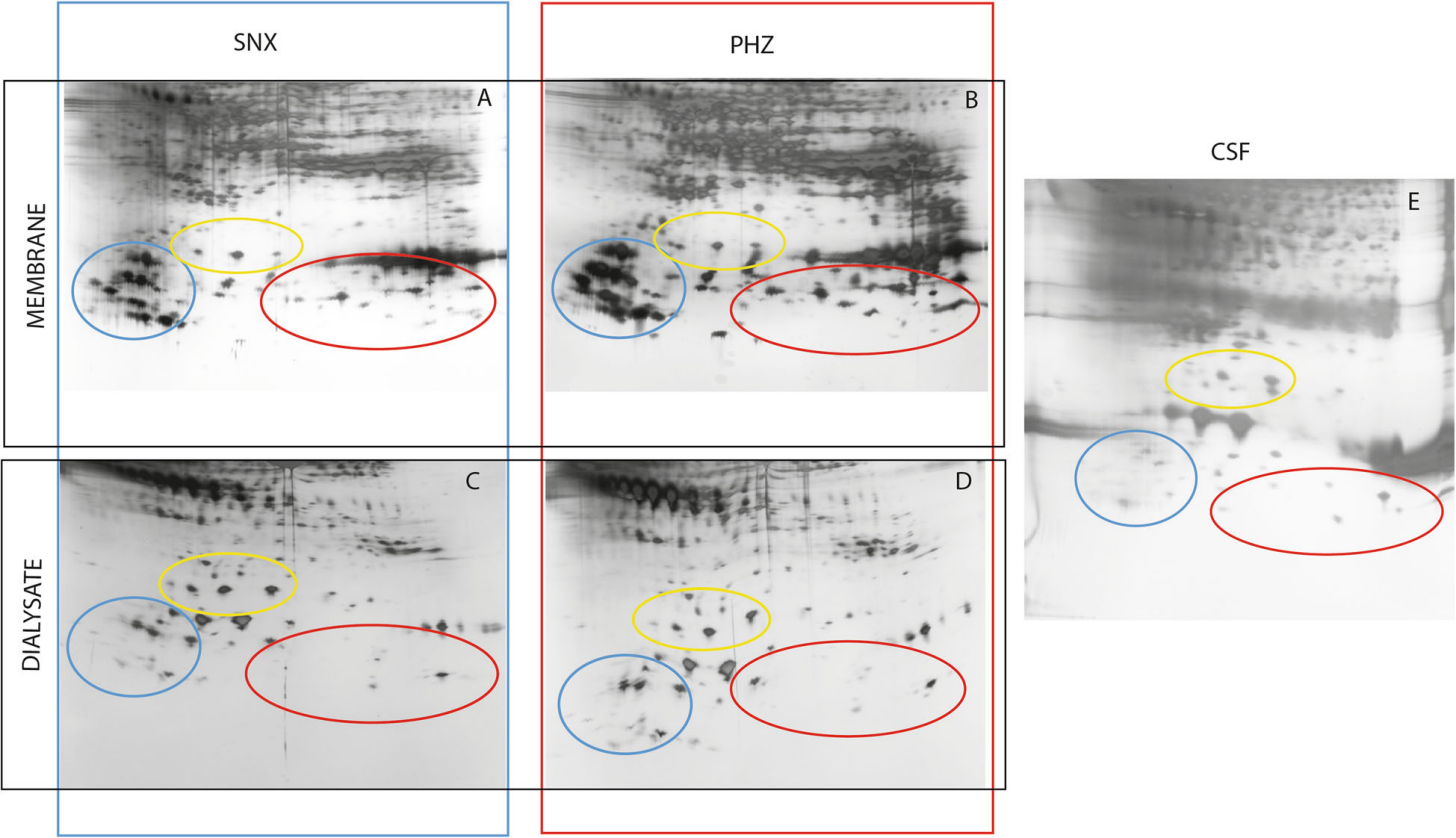
Protein patterns of microdialysis catheter membrane after insertion in (**a**) seemingly normal cortex and (**b**) perihemorrhagic zone. The dialysis samples from (**c**) seemingly normal cortex, (**d**) perihemorrhagic zone and (**e**) cerebral spinal fluid. Marked areas indicate visual protein pattern differences. Images are cropped for clarity. The full gel image is available as Supplemental Figure 1

2-DE analysis revealed a characteristic protein spot pattern for each sample compartment with the highest number of spots in CMD membrane samples (mean 448.2 +/− 56) compared to CSF (mean 352.3 +/− 73) and dialysate samples (mean 301.0 +/− 28). A representative electrophoregram for CSF, dialysate and CMD membrane for one patient is shown in Fig. 2. There was a clear visual difference in the pattern of proteins with molecular weight < 50 kDa (circled areas in Fig. 2) so we focused on identifying protein spots in this area.

### Distinct differences in protein expression in CSF, microdialysate and CMD membrane

**Fig. 3:**
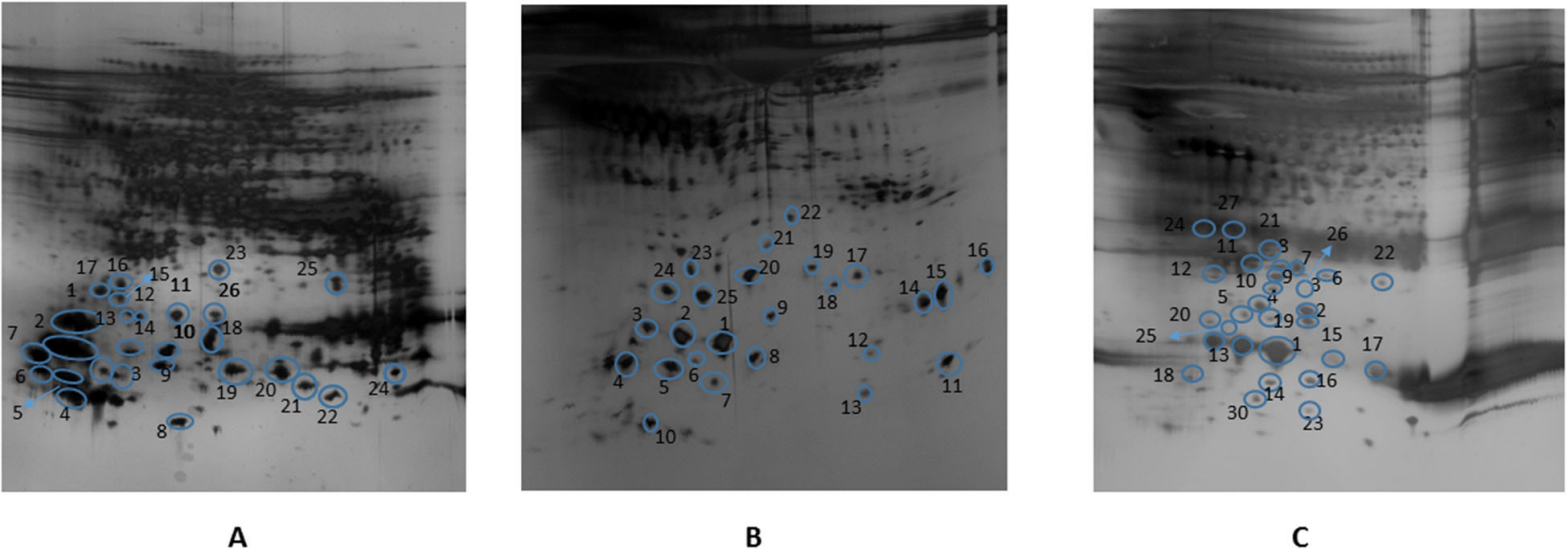
Two-dimensional electrophoregram for (**a**) catheter membrane, (**b**) dialysate and (**c**) CSF samples. Marked numbers refer to the identified spot numbers in Table 2

**Fig. 4:**
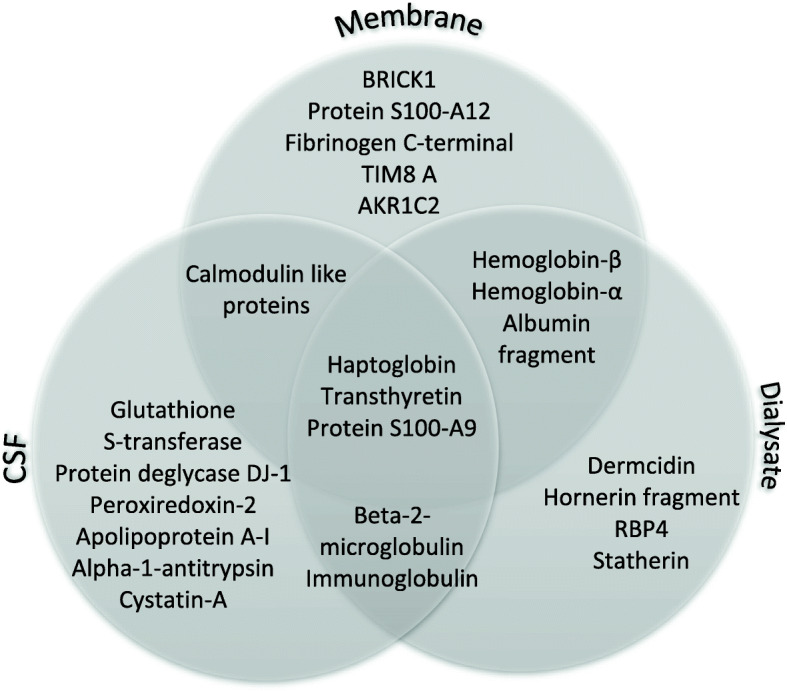
Venn diagram showing which proteins were found in each compartment and illustrating overlap. Abbreviations: TIM8 A = Mitochondrial import inner membrane translocase subunit TIM8 A; AKR1C2 = Aldo-keto reductuase family 1 C2; RBP4 = Retinol Binding Protein 4

**Fig. 5:**
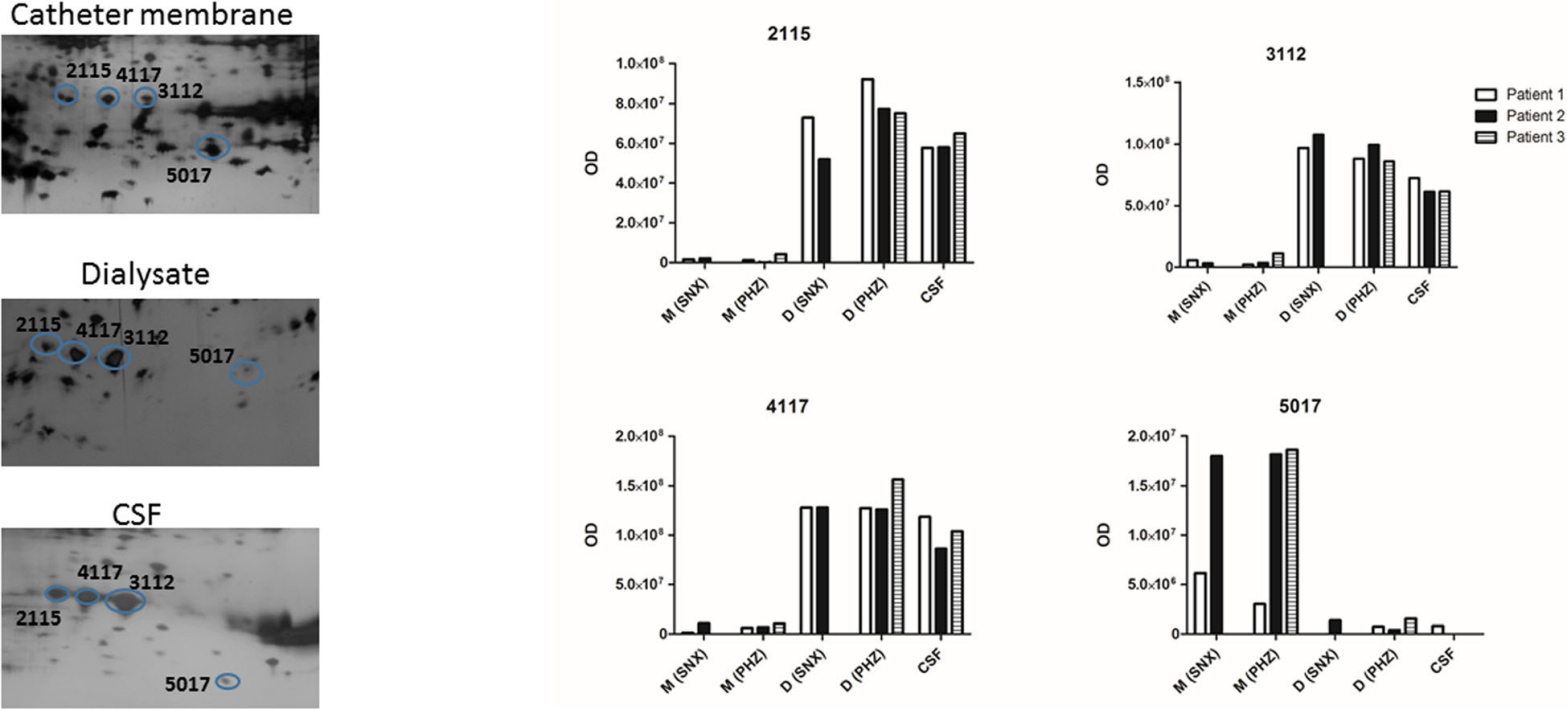
Quantitative comparison of selected protein spots in catheter membrane, dialysate and CSF samples. The numbers of the marked spots on the gels refer to the spot numbers in the diagram. The diagram shows optical density (OD) for each protein spot in catheter membranes from seemingly normal cortex (M-SNX), catheter membranes from perihemorrhagic zone (M-PHZ), dialysate from seemingly normal cortex (D-SNX) and from perihemorrhagic zone (D-PHZ) and CSF. Spot numbers 2115, 3112 and 4117 were identified as transthyretin (P02766). Spot number 5017 was identified as mutant hemoglobin beta chain, fragment (Q6V0K9). The images are cropped from original gel-images that are displayed in full in Fig. 3. Abbreviations: OD = optical density; M = membrane; D = dialysate; CSF = cerebrospinal fluid; PHZ = perihemorrhagic zone; SNX = seemingly normal cortex

Distinct groups of protein spots could be observed on the CMD membrane gels which were totally missing from both CSF and microdialysate gels (Figs. 2 and 3). These protein spots were chosen for further analysis and identified by mass spectrometry (Table 2, Fig. 3). Spots of interest were excised, 68 spots from membrane gel and 62 from the CSF-gel, destained, digested by trypsin and analyzed by mass spectrometry. Results of identified spots on the dialysate gels have been previously published [13]. Some of these results are included in Table 2 of this current study for clarity. Twenty-five proteins from the membrane gel and 30 proteins from the CSF gel were identified. Results were dominated by high abundant plasma proteins such as, hemoglobin, apolipoproteins and haptoglobins, but there were also less abundant proteins specifically present only on the membrane and not in CSF or in microdialysate including BRICK1, mitochondrial import inner membrane translocase subunit TIM8 A, and Aldo-keto-reductase family 1 member C2 (Table 2; Fig. 4). Furthermore, there were distinct quantitative differences between the three compartments (Fig. 5), although in this pilot study the sample size was not large enough to perform quantitative analysis of differences.

**Table 2:** Identified proteins in samples extracted from CMD membrane (M), CSF (C) and dialysate (D)

*Spot number*	*Accession number*	*Name of protein*	*Sequence covering (%)*	*Number of peptides*	*Theoretical pI/Mw*
M1	P02766	Transthyretin	49.0	5	5.5/15887
M2	P02766	Transthyretin	39.5	3	5.5/15887
M3	P80511	Protein S100-A12	16.3	2	5.3/10575
M4	P02766	Transthyretin	48.3	4	5.5/15887
M5	P02766	Transthyretin	39.5	3	5.5/15887
M6	P02766	Transthyretin	39.5	3	5.5/15887
M7	P02766	Transthyretin	39.5	3	5.5/15887
M8^#^	119598216^a^	Calmodulin-like 4, isoform CRA c	30.0	3	5.9/13661
M9^#^	Q8WUW1	BRICK1	40.0	3	5.4/8745
M10^#^	O60220	Mitochondrial import inner membrane translocase subunit Tim8 A	64.9	3	5.1/10998
M11	P02766	Transthyretin	68.7	7	5.5/15887
M12	P02766	Transthyretin	48.3	4	5.5/15887
M13	P02766	Transthyretin	39.5	3	5.5/15887
M14	P00738	Haptoglobin alpha chain	17.5	4	5.6/15945
M15	P02671	Fibrinogen C-terminal	48.4	12	4.6/27292
M16	P02766	Transthyretin	63.9	5	5.5/15887
M17	P00738	Haptoglobin	32.5	9	6.3/276265
M18^#^	P06702	Protein S100-A9	57.9	6	5.7/13242
M 19^#^	E9NGZ5	Hemoglobin beta globin chain (Fragment)	93.3	7	5.9/11494
M20^#^	Q6V0K9	Mutant hemoglobin beta chain (Fragment)	94.3	8	6.2/11474
M21^#^	Q6V0K9	Mutant hemoglobin beta chain (Fragment)	76.2	6	6.2/11474
M22^#^	Q9BWU5	Mutant hemoglobin beta chain (Fragment)	45.7	4	6.5/11501
M23^#^	C9JKR2	Serum albumin (fragment)	19.7	6	6.0/47288
M24^#^	Q9BX83	Hemoglobin alpha 1 globin chain (Fragment)	52.0	4	7.1/10710
M25^#^	P52895	Aldo-keto reductase family 1 member C2	14.9	4	7.1/36736
C1	P02766	Transthyretin	49.0	5	5.5/15887
C2	P00738	Haptoglobin	32.8	10	6.3/276265
C3	P00738	Haptoglobin	30.8	11	6.3/276265
C4	P00738	Haptoglobin	30.6	9	6.3/276265
C5	P02766	Transthyretin	32.7	3	5.5/15887
C6	Q99497	Protein deglycase DJ-1	42.3	6	6.3/19891
C7	P01876	Ig alpha-1 chain C region	21.5	6	6.0/37654
C8	P09211	Glutathione S-transferase P	28.6	4	5.4/23356
C9	P09211	Glutathione S-transferase P	21.4	3	5.4/23356
C10	P32119	Peroxiredoxin-2	32.3	6	5.6/21892
C11	P02647	Apolipoprotein A-I	42.3	10	5.4/30777
C12	P02647	Apolipoprotein A-I	42.3	10	5.4/30777
C13	P02766	Transthyretin	63.9	6	5.5/15887
C14	P06702	Protein S100-A9	38.6	5	5.7/13242
C15	P06702	Protein S100-A9	56.1	6	5.7/13242
C16	P00738	Haptoglobin	14.3	3	6.3/276265
C17	P01009	Alpha-1-antitrypsin; Short peptide from AAT	9.6	3	5.3/46736
C18	P02766	Transthyretin	40.1	4	5.5/15887
C19	P00738	Haptoglobin	26.1	11	6.3/276265
C20	P00738	Haptoglobin	20.0	9	6.3/276265
C21	P02647	Apolipoprotein A-I	50.6	13	5.4/30777
C22	P01876	Ig alpha-1 chain C region	21.5	6	6.0/37654
C23	Q9NZT1	Calmodulin-like protein 5	9.6	2	4.3/15751
C24	P01009	Alpha-1-antitrypsin;Short peptide from AAT	10.3	6	5.3/46736
C25	P01040	Cystatin-A	36.7	4	5.4/11006
C26	P32119	Peroxiredoxin-2	26.8	5	5.6/21892
C27	P02647	Apolipoprotein A-I	67.0	18	5.4/30777
C28	P02647	Apolipoprotein A-I	59.2	15	5.4/30777
C29	P02647	Apolipoprotein A-I	18.0	5	5.4/30777
C30	P61769	Beta-2-microglobulin form pI 5.3.	16.8	2	5.3/11618
D1	P02766	Transthyretin	55.8	6	5.5/15887
D2	P02766	Transthyretin	55.1	5	5.5/15887
D3	P02766	Transthyretin	39.5	3	5.5/15887
D4	P02768	Albumin fragment	3.8	2	6.0/ 47,288
D5	P00738	Haptoglobin	6.4	3	6.3/276265
D6	P06702	Protein S100-A9	26.3	2	5.5/15887
D7	P01877	Ig alpha-2 chain C region (Ig like-2 domain)	10.2	3	6.1/10095
D8	P02768	Albumin fragment	28.9	15	6.0/ 47,288
D9	P00738	Haptoglobin	6.4	3	6.3/276265
D10	Q86YZ3	Hornerin fragment	6.4	4	4.8/9565
D11	Q86YZ3	Hornerin fragment	6.4	4	4.8/9565
D12	P61769	Beta-2-microglobulin	16.8	2	5.3/11618
D13	P81605	Dermcidin	10.0	1	6.1/ 11,283
D14	P68871	Hemoglobin subunit beta	43.5	3	6.8/ 15,867
D15	P68871	Hemoglobin subunit beta	47.5	2	6.8/ 15,867
D16	P69905	Hemoglobin subunit alpha	36.6	2	8.7/ 15,126
D17	P02768	Albumin fragment	10.0	3	6.0/ 47,288
D18	P81605	Dermcidin	20.0	2	6.1/ 11,283
D19	P00738	Haptoglobin	8.1	8	6.3/45200
D20	P00738	Haptoglobin	6.4	3	6.3/45200
D21	P02808	Statherin	54.8	1	8.0/7304
D22	P02768	Albumin fragment	11.3	7	6.0/ 47,288
D23	P02753	Retinol-binding protein 4	24.9	5	5.3/ 21,071
D24	P00738	Haptoglobin	6.2	2	6.3/45200
D25	P00738	Haptoglobin	6.2	2	6.3/45200

Spot number refers to numbers in Fig. 5. Abbreviations: pI = isoelectric point; Mw = molecular mass. ^a^indicates protein accession number according to NCBInr. Proteins were identified by MALDI-TOF (indicated by #) or LC-MS/MS (no #)

## Discussion

This study is the first to report on the pattern of protein adsorption to CMD membranes following implantation in human brain tissue. It is also the first to compare this pattern to that of CSF and microdialysate; two compartments commonly sampled for biomarker discovery. Our results demonstrate that proteins adsorb to the CMD membranes and thus may not be present in the microdialysate or CSF samples.

Distinct proteins were found on the CMD membrane which were not identified in the dialysate or in CSF. Among these were BRICK1 which is required for cell proliferation and cell transformation, directional migration and its downregulation is associated with invasive growth in various tumor cell types [18]. Protein S100-A12 is a small calcium binding protein of the S100-family, which is excreted mainly by neutrophil granulocytes and to some extend by monocytes. It plays a role in a variety of extracellular activities part of the innate immune responses including chemotaxis and activation of intracellular signalling cascades leading to cytokine production [19]. Fibrinogen is an important part of the coagulation cascade and can enter and deposit in the brain following vascular injury or compromise of the blood-brain-barrier. It has been recently shown to have a pleotropic role in the CNS including activation of inflammation, induction of scar formation, promotion of cognitive decline and inhibition of repair [20, 21]. Mitochondrial import membrane translocases have an implied role in several neurological disease conditions [22–26]. Another protein found exclusively on the CMD membrane was Aldo-keto reductase 1C2 (AKR1C2) shown previously to be increased in the late stages of Alzheimers Disease [27]. These proteins would thus not have been detected had only the microdialysate or CSF been analysed.

In recent years CMD has been used as part of NCC monitoring and to study biomarkers of various neurological diseases in particular traumatic brain injury [28–30], gliomas [31, 32], ischemic stroke [1], intracerebral hemorrhage [33] and subarachnoid hemorrhage [34, 35]. CMD sampling for subsequent proteomic biomarker analysis has advantages, such as capture of proteins near the site of origin in the extracellular space, and no effect of dilution as is the case in CSF or plasma [28]. But there are well known challenges to the microdialysis method including its focal resolution, limited time resolution, small sample volume [36], a tendency to elicit an inflammatory response [37] and variations in relative recovery [38, 39], which is in part due to protein adsorption. Such protein adsorption on CMD membranes implanted in patients has been poorly described to date and needs to be further explored as illustrated in our present pilot study.

Ultrafiltration is another technical issue to consider when using high-molecular-weight cut-off CMD catheters (100 kDa). Ultrafiltration means that fluid escapes from the perfusate into the sampling tissue thereby changing the environment immediately surrounding the CMD catheter [2, 36, 40]. To counteract ultrafiltration colloids such as albumin can be added to the perfusate as was clinical routine in our department during the study period. However, albumin is known to adsorb to membrane surfaces and may in turn affect the recovery of other proteins. This was indirectly shown in a previous study using CMD in a NCC setting where the addition of albumin to the perfusate was found to be necessary in order to extract amyloid-β (Aβ) in the microdialysate. The authors argue that this was presumably due to Aβ adhering to the CMD membrane and tubing in the absence of albumin [41]. It is plausible that the addition of albumin to the perfusate also in the present study has affected the pattern of protein adsorption to the CMD membrane, and use of a different perfusate could result in a different pattern. Therefore the pattern of protein adsorption to CMD membranes cannot be assumed to be the same in different study protocols as protein adsorption will be affected by a number of factors, of which perfusate composition is one. This is why we recommend analyzing membrane adsorbed proteins when sampling macromolecules using CMD.

Modifications can be made to either the membrane or the perfusate in order to increase relative recovery of macro molecules. Modifications to the perfusate include the addition of antibodies with high affinity for a specific compound of interest which can increase its relative recovery as has been done with cytokines [42]. This method, however, is not relevant when the aim is to sample all proteins present. Previous in vitro and preclinical studies show decreased protein adhesion to both CMD membrane and tubing following catheter modifications using triblock copolymers such as Pluronics 127® [6, 43]. However, to date these modifications have only been used in vitro or in preclinical studies. Certain proteins of interest may display particular tendencies to adhere to surface materials, such as for example Aβ protein and modifications of membrane and tubing may be useful in studies focused on such particularly ‘sticky’ proteins. As this present study shows proteins have differing tendency to adsorb to the membrane. An awareness of this is recommended when using CMD for sampling macromolecules.

It has been suggested that adsorption of proteins to the membrane surface is the first step in a potential foreign body reaction elicited by catheter implantation [44]. Such a foreign body reaction has not been fully characterized following implantation in human brain, however, several preclinical studies have shown an immediate traumatic response in brain tissue following CMD catheter implantation [12]. Furthermore, one study using scanning electron microscopy demonstrated buildup of cellular debris on CMD membranes implanted in human brain tissue [40]. This cellular debris is presumably preceded by protein adsorption to the membrane surface, and characterization of such protein adsorption can plausibly give more insight also into the foreign body reaction.

In a recent study of ICH patients, using a similar study design we found a difference in proteins expressed in the perihemorrhagic zone (PHZ) compared to seemingly normal cortex (SNX) [13]. In light of the results of this present study it is likely that several proteins evaded detection by adsorbing to the CMD membranes. Plausibly, a paired catheter design involves a similar pattern of protein adsorption to both catheters, but this cannot be guaranteed. Factors such as pH, rate of diffusion, and brain tissue edema may differ locally in the tissue and thus protein adsorption to CMD membranes could be different in two catheters placed in different regions of the brain. This can only be adequately characterized by analyzing the membranes.

Limitations of the present study include the small sample size, which precludes any comparisons or correlation with clinical parameters such as outcome or medical or surgical complications. However, the study design is complex and the obtained data is highly novel. Furthermore, the present study cannot determine the effect of time on protein adsorption, and future studies should aim to elucidate the time course of protein adsorption to CMD membranes in brain tissue as this may cause relative recovery to change over time. Another limitation of this study is that only proteins between 10 and 50 kDa could be investigated. There might be proteins of interest with a molecular weight higher than 50 kDa that are masked by the high amount of albumin. Future studies focusing on improved albumin depletion or using another perfusion fluid to obtain better resolution for protein separation are warranted.

The present study is, to the best of our knowledge, the first to characterize protein adsorption to CMD membranes after implantation in human brain tissue. Previous studies have described protein adhesion to membranes in animal studies or in vitro studies [7, 11]. One such in vitro study described protein adsorption to two microdialysis membranes after dialysis of a sample matrix of ventricular CSF. The study showed that 50% of proteins found on the CMD membrane were not identified in the sample matrix pointing to methodological limitations. This study used on-surface tryptic digestion of proteins on the membrane prior to denaturation, followed by a bottom-up approach for protein identification using LC-MS/MS. Technical issues may explain the low overlap of proteins between the CMD membrane and the sample matrix. To avoid failure of proteins to desorb from the CMD membrane in the present study we employed a protocol previously determined by our group, used on membranes implanted in human muscle tissue (unpublished data), and calibrated for optimal protein elution.

By showing particular differences in the proteome profile of the three compartments, CMD membrane, microdialysate and CSF, our results emphasize the need to analyze protein adsorption to CMD membranes in addition to microdialysate protein content and highlight the need to interpret analysis results of proteins sampled by CMD with caution. Modifications to both catheter membranes and perfusion fluid may enable improved recovery of large molecules. Our results emphasize the need to analyze protein adsorption to CMD membranes in microdialysis research, particularly when using CMD for biomarker discovery.

## Conclusion

Information on protein content in interstitial fluid is inadequately described by analyzing only the microdialysate, since proteins present may adsorb to and be found only on the CMD membrane. These proteins may play an important role in understanding the pathophysiology of the studied disease, in this case intracerebral hemorrhage (ICH), and failure to identify them in the dialysate samples may lead to incorrect conclusions. The adsorbed proteins may also alter the function of the membrane and thereby affect the dialysate composition and influence the concentration of the proteins in the microdialysate. When using CMD to sample proteins from human brain tissue we recommend analysis of protein adsorption to the membranes in addition to in the microdialysate samples.

## Supplementary information

**Additional file 1: Supplemental Figure 1.**

## Data Availability

The datasets generated during and/or analyzed during the current study are available from the corresponding author on reasonable request.

## Abbreviations

2-DE: Two-dimensional gel electrophoresis
CMD: Cerebral microdialysis
CSF: Cerebrospinal fluid
EVD: External ventricular drain
ICH: Intracerebral hemorrhage
MD: Microdialysis
NCC: Neurocritical care
PHZ: Perihemorrhagic zone
SNX: Seemingly normal cortex
