# Supplementary figures and images for: Proteomic investigation of protein adsorption to cerebral microdialysis membranes in surgically treated intracerebral hemorrhage patients - a pilot study
Source: Proteome Sci. 2020 Jul 25;18:7. doi: 10.1186/s12953-020-00163-7 (PMC7382826; doi:10.1186/s12953-020-00163-7)

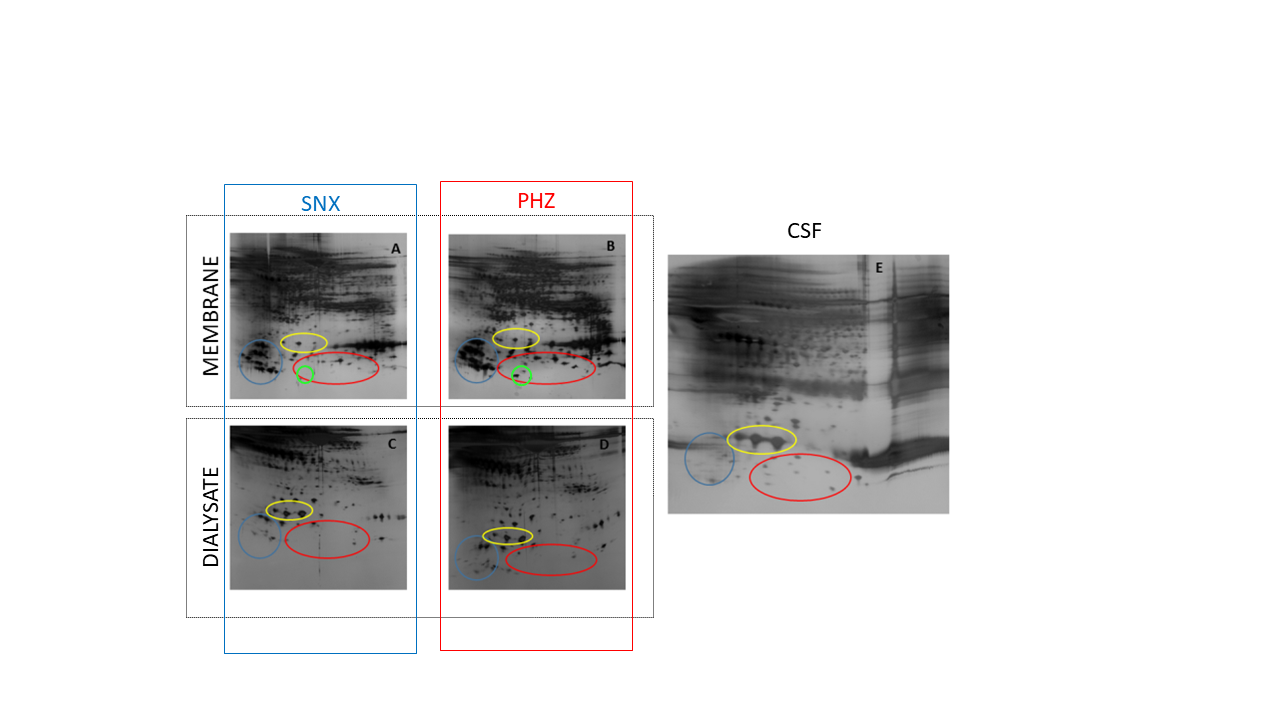

Supplement: Supplementary file 1 — Additional file 1: Supplemental Figure 1. [file 12953_2020_163_MOESM1_ESM.tif]
